# Mobility, Expansion and Management of a Multi-Species Scuba Diving Fishery in East Africa

**DOI:** 10.1371/journal.pone.0035504

**Published:** 2012-04-17

**Authors:** Hampus Eriksson, Maricela de la Torre-Castro, Per Olsson

**Affiliations:** 1 Department of Systems Ecology, Stockholm University, Stockholm, Sweden; 2 School of Biological Sciences, The University of Sydney, Sydney, New South Wales, Australia; 3 Stockholm Resilience Centre, Stockholm University, Stockholm, Sweden; Leibniz Center for Tropical Marine Ecology, Germany

## Abstract

**Background:**

Scuba diving fishing, predominantly targeting sea cucumbers, has been documented to occur in an uncontrolled manner in the Western Indian Ocean and in other tropical regions. Although this type of fishing generally indicates a destructive activity, little attention has been directed towards this category of fishery, a major knowledge gap and barrier to management.

**Methodology and Principal Findings:**

With the aim to capture geographic scales, fishing processes and social aspects the scuba diving fishery that operate out of Zanzibar was studied using interviews, discussions, participant observations and catch monitoring. The diving fishery was resilient to resource declines and had expanded to new species, new depths and new fishing grounds, sometimes operating approximately 250 km away from Zanzibar at depths down to 50 meters, as a result of depleted easy-access stock. The diving operations were embedded in a regional and global trade network, and its actors operated in a roving manner on multiple spatial levels, taking advantage of unfair patron-client relationships and of the insufficient management in Zanzibar.

**Conclusions and Significance:**

This study illustrates that roving dynamics in fisheries, which have been predominantly addressed on a global scale, also take place at a considerably smaller spatial scale. Importantly, while proposed management of the sea cucumber fishery is often generic to a simplified fishery situation, this study illustrates a multifaceted fishery with diverse management requirements. The documented spatial scales and processes in the scuba diving fishery emphasize the need for increased regional governance partnerships to implement management that fit the spatial scales and processes of the operation.

## Introduction

Increasing global demand and mobility of markets are strong drivers for unsustainable fisheries in the world [1], [2]. The depletion of near-shore fishing grounds and technological development has driven spatial expansion [3], and fishing down marine food webs [4], predominantly to serve affluent markets [5]. Compared to finfish fisheries, invertebrate fisheries have expanded multitudes faster than science and policy to support sustainable fishing [6]. For example, the international trade with sea urchins for the Japanese market illustrates a global destructive expansion in marine resources harvesting, and a failure of existing governance arrangements to deal with such problems [7]. Illegal, unregulated and unreported (IUU) fisheries constitute some of the biggest governance challenges to sustainable fisheries [8]–[10], for example approximately 40% of the West African total catch may be illegal or unreported [11]. The conduct shaping expansion of marine resource extraction, moving to new fishing grounds with depletion of old, or to prospect for new ones, has been referred to as a roving bandit behavior [7], following Mancur Olson's description of anarchic roving bandits that lack incentives to govern and preserve [12]. Roving bandits' conduct is defined by evasion of norms and rules or taking advantage of missing institutions, in favor of personal profits. In the context of fisheries, this conduct comes at a cost of deteriorated ecological resilience of marine ecosystems and has negative consequences for trans-generational equity through degraded baselines for coming generations.

**Figure 1:**
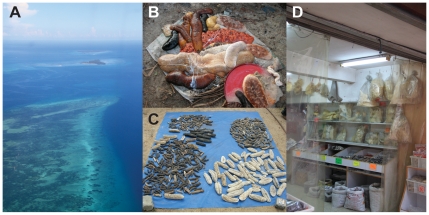
Sea cucumbers from ecosystem to market. a) Aerial view over Ukombe reef, Kwale and Pungume Islands, which were frequently visited fishing grounds by scuba divers in this study, b) Scuba diving fishers catch (*Actinopyga miliaris, Bohadscia subrubra, B. vitiensis, Holothuria fuscogilca, H.isuga, H. lessoni, Theleonota ananas, T. anax*) in Mkokotoni, Zanzibar, c) sun drying of various varieties of bêche-de-mer in Mtoni, Zanzibar, d) Shark-fins and bêche-de-mer for sale on Queen's Road West in Hong Kong. Photos: Hampus Eriksson.

The dried seafood market in Asia is an example of a trade system where resources such as dried sea cucumber (known as bêche-de-mer or trepang) [13] and dried shark fin [14] are harvested in distant marine environments to serve a proximal market. The sea cucumber fishery has a similar development pattern to that of the spatial expansion of the sea urchin fishery [7], albeit on a longer time-scale [15], [16]. Harvest and trade of sea cucumbers is now occurring in most of the tropical world [17], having expanded from its origin in the central Indo-Pacific where it has been active since at least the 1700s [15], [18]. Tropical sea cucumber fisheries are complex multi-species and multi-user fisheries [19] embedded in global trade networks with several actors operating at different levels, i.e., from local fishers, via middlemen and multinational networks to consumers predominantly in China [20] (Fig. 1). Existing institutions have generally failed in securing sustainable harvesting of this resource, which has resulted in a destructive boom-and-bust pattern and a high level of IUU fishing [21]. Many sea cucumbers that are targeted in tropical fisheries are broadcast spawners that reproduce on an infrequent annual basis [22]. This reproductive strategy makes them vulnerable to overfishing, which has commonly reduced population densities to levels where the Allee effect restrains population growth, sometimes leading to local extirpation [23]. This raises concerns about the ecological impact of these fisheries because sea cucumbers recycle organic material, thereby contributing to ecosystem productivity [24], [25]. They also ingest large quantities of carbonate sands [26], which partially dissolve in the gut and influence the CaCO_3_ budget on coral reefs [27].

This study focuses on the scuba diving fishery and trade in Zanzibar (Unguja Island) in East Africa, which is a part of the islands sea cucumber fishery. Eriksson et al. [28] identified the general features of this fishery and the existence of a scuba diving fishery. The Department of Fisheries and Marine Resources (DFMR) is responsible for the management of the fishery. While there are a number of broad legislations in place that indirectly concerns the sea cucumber fishery, such as annual licenses for fishing boats or exporting marine products, no fishery-specific management plan exists. There are some villages that have informal institutions, but these are not widely known or followed. This management situation has not been successful, or adaptive to declining trends, and existing institutions do not sufficiently govern the conduct of fishers or actors in the trade. For example, the fishery is only formally regulated through a size restriction (10 cm length), which is neither communicated nor enforced, making it a blunt management tool. In addition, the length is ecologically irrelevant as it is smaller than the known sizes of sexual maturity [22]. Moreover, authorities do not monitor the fishery other than for revenue purposes from exports, so the fishery is *de facto* operating without regulation or control. Due to the lack of management Eriksson et al. [28] argue that the scuba diving fishery had evolved following the path of unregulated sea cucumber fisheries illustrated by Friedman et al. [29], where the depletion of easy-access high-value stocks had resulted in a progression in the collection method from village scale near-shore collection through gleaning or breath hold diving, to a more mobile boat-operated scuba diving enterprise. Understanding the patterns and processes associated to this multifaceted fishery situation is important because there may be different management requirements. In this context, recent large-scale reviews of sea cucumber fisheries [19], [21], [30], although highlighting the problem of complexity and IUU fishing, focus on generic improvements to a simplified fishery situation. This study complements these reviews through an in-depth analysis of the multifaceted fishery context in Zanzibar.

Diving for sea cucumbers using underwater breathing devices has been described as a “human and environmental disaster”, due to the extreme exposure to accidents and indiscriminate harvesting [31], [32]. Despite that scuba diving for sea cucumbers has been observed to commonly occur in the Western Indian Ocean (WIO) (e.g., Kenya, Madagascar, Seychelles and Tanzania [33], and Zanzibar [28]), and that presence of this activity generally indicates an unsustainable fishery [29], no systematic studies have been done focusing on this category of mobile fishing. This is worrying because the fishery is not only extremely dangerous for the individual fisher, but the geographic scale and mode of the operation is unknown making governance and informed management responses difficult. Therefore, we here address these knowledge gaps by studying the scuba diving fishery in Zanzibar through three conceptual parts; (i) the fishery operation to determine the geographic scales of exploitation and trade, (ii) catch and economic patterns in the operation to examine the processes in fishers' strategies, and (iii) social aspects associated with the scuba diving fishers, to assess their role in the trade system and discuss social pros and cons.

## Methods

**Figure 2:**
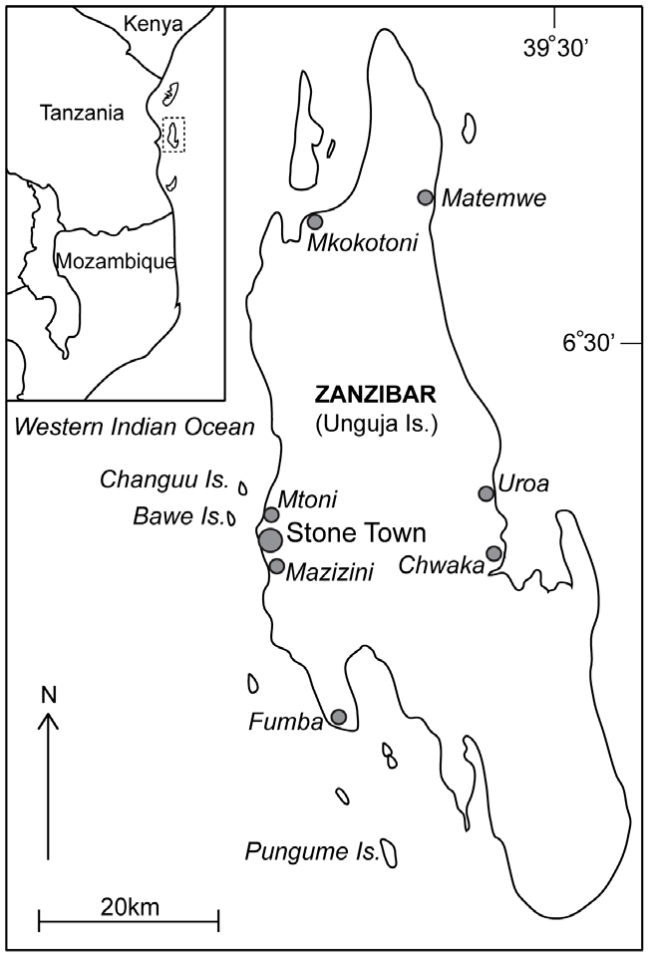
Map of Zanzibar (Unguja Island). Villages where the scuba diving fishery was studied are indicated with grey dots.

Four different methods (i.e., interviews, discussions, participant observations, and boat monitoring) were used to approach the three conceptual parts of the scuba diving fishery (i.e., fishery operation and geographic scale, catch and economic patterns, and social aspects) (Table 1). The villages selected for the study were Mkokotoni, Mazizini, Mtoni, Fumba, Matemwe, Chwaka and Uroa (Fig. 2). The selection of villages was based on previous research [28] and the local knowledge of the scientists working at the Institute of Marine Science, University of Dar-es-Salaam (IMS). Both the researchers and two trained local Zanzibar staff members from IMS were active in data collection.

**Table 1:** Methods used in the study.

Method	Fishery operation and geographic scale	Catch and economic patterns	Social aspects
**Interviews**			
Focus group interviews and in-depth interviews	X	X	X
**Discussions**			
Informal discussions with diving fishers	X		X
**Observations**			
Observations of catch and organization	X	X	X
**Boat monitoring**			
Systematic boat monitoring of divers boats	X	X	

Outline of methods used to analyze the three conceptual parts studied in the scuba diving fishery in Zanzibar.

### Ethical statement

The Department of Systems Ecology Review Board (Stockholm University) has approved the study. Interviews with participants in this study were analyzed anonymously. Participants disclosed of their name on a voluntary basis after being informed of the purpose of the study and that their answers would remain anonymous. Verbal consent was given by participants after being informed of the purpose and analysis of the study. Only those participants giving consent were interviewed. Given that the participants were anonymous, and that the purpose of the analysis did not encompass elements that would compromise personal integrity and safety or hinge on socially constructed groupings (e.g., race, religion or gender), no further written consent or ethics approval was sought for the study.

### Interviews

Focus group interviews were conducted following the description by Bunce et al. [34]. This is a semi-structured interview method that permits interaction and discussion among group participants. In pilot interviews, individual divers were reluctant to one-on-one interviews so this method was adopted because it facilitates group participation, which was considered more appropriate to the situation. Seven focus group interviews were done (one in each of the villages). Interviews did not aim to support a quantitative comparative analysis between villages because this type of method has limited scope for such analysis. Instead, the method was chosen to provide a description of the fishery situation by building on the shared experiences and perceptions of divers and other participants involved in the activity. Interviews were performed with groups of two to five key informants (middlemen, divers, monitoring agents and elder retired divers or fishers) during approximately two to three hours in each village. Interviews followed a specific thematic guideline (Text S1). The interviewer introduced each theme and the respondents were able to speak freely and discuss each subject. Fishing grounds were identified by name in interviews or by participants pointing at a map. The same thematic guideline was also used to perform in-depth interviews [35] with the village monitoring agents (*Bwana dikos* in Swahili) from the Department of Fisheries and Marine Resources (DFMR) responsible for monitoring village fishing activities [36]. Five in-depth interviews were performed with monitoring agents. All interviews were performed by the same research assistant from IMS having local knowledge about fishing villages and with Swahili as mother tongue. On the same day that interviews were performed all information was checked and transcribed. Each village was approached in the following way: During the first day the local research assistant visited the village to perform observations and contact key informants. The whole day was spent in the village making observations. During the second day the focus groups interviews took place and in-depth interviews were done during a third day. In some villages, an extra one-day visit was done to clarify issues, or to visit important facilities such as the divers' decompression chamber in Matemwe or the villages' processing facilities. In addition, the doctor in charge of the decompression chamber was interviewed. Interviews were analyzed addressing the research objectives (e.g., fishery operation and geographic scale, catch and economic patterns, and social aspects) and crosschecked for the existence of data in all villages [37].

### Discussions and participant observations

Additional information was collected through informal discussions and participant observations. This data was collected by the researchers, predominantly at landing sites, while living in villages Mkokotoni, Fumba and Uroa, and having continuous interaction with the actors in the fishery over the length of the stay (approximately 20 days per village). Informal discussion included asking about how the equipment was working, if catch was good, and which fishing grounds were visited. Answers to these questions indicated important aspects of the operation such as safety issues, spatial scales, organization, and trends in catch. Observations were used predominantly in the transaction phase between fishers and middlemen and what catch was being traded. The observations were then followed up by questions such as what was paid for and if that was a good price. Observations and discussions complemented the interviews in creating a comprehensive view of the interaction among actors in the fishery, particularly relating to the divers' organization and their relationship towards the other actors in the fishery. The IMS assistant translated and facilitated discussions. In addition, the researchers frequently interacted with a retired diver who was a high member of the fishing committee in Mkokotoni and a middleman engaged to purchase catch in Mkokotoni on behalf of a Chinese exporter in Mtoni. Both participated in the study as key-informants providing detailed descriptions on trade and divers organization as well as regarding trends in the fishery. The middleman also permitted the researchers to visit the exporters processing facility Mtoni.

### Boat monitoring

Five boats operating out of Mtoni, Mazizini and Fumba, visiting fishing grounds Bawe, Changuu and Pungume (Fig. 2), were systematically monitored for ten days in December 2009 (NE Monsoon) and for ten days in May 2010 (SW Monsoon). Fifty-eight trips were recorded, but three data points were omitted due to unclear catch value for some species. The strategy to divide the monitoring between the two monsoon seasons was because previous work [23] had indicated that fishers were more active during December, due to the more favorable weather improving seafaring and visibility. These two seasons are hereafter referred to as high (December) and low (May) season. Boats operating in other villages, or targeting other fishing grounds, which we did not have the resources to sample may differ in catch or strategies. The trained staff member at the IMS carried out the monitoring by documenting the landed catch, effort, and value as paid to fishers.

The catch value data were not balanced for visited fishing grounds or boats over the two monitored seasons, and so did not permit a nested analysis. A hierarchical partitioning approach was therefore chosen to perform a goodness of fit analysis of the predictor variables “season” (high or low), “boats” (five boats) and fishing “grounds” (three fishing grounds) in R 2.9.2 as described by Logan [38]. This is a multiple regression method that calculates goodness of fit measures on how much of the variation in catch value that each of the predictor variables explains. The variables' independent contribution was tested using a randomization test performed with 1,000 permutations, which produces a Z-score and statistical significance based on upper 0.95 confidence limit [38].

The species-specific catch per unit effort (CPUE) and value per unit effort (VPUE) in pieces of catch or in Tanzania Shilling (TSZ) per trip was calculated (n = 55). For each trip the number of pieces (per species) and their value was divided by the number of divers and the time spent fishing for that trip. These two measurements were used to explore abundance and value patterns in catch. Export data was collected from DFMR.

## Results

### Fishery operation and geographic scale

The median number of divers per team (boat) was five, but the median number of team members on-board was seven (including non-diving boat operators). In interviews team size ranged from four to ten members. The median time spent fishing was four hours (not including travel time to fishing ground). The estimated number of scuba divers in the studied locations was about 200–300, but this was difficult to estimate because the number varied over the year with more divers participating during high season (Table 2). The center of the diving fishery was in Mtoni, where at least four exporters operated. Presence of migrating divers was mentioned in all studied villages. Divers were either resident in, and operated out of, the urban areas near the exporters' location (i.e., Mtoni and Mazizini), or they lived in, and operated out of, villages around the island (i.e., Mkokotoni, Matemwe and Fumba). Divers also travelled across the island from the urban areas near Stone Town to set up camp in Uroa or Chwaka during favorable monsoon on the east coast. This was also mentioned as a good area to find the high value teatfish varieties (e.g., *Holothuria nobilis*), exemplifying that strategies take into account weather and resource availability. For the high season, fishers traveled to the Mtoni area to fish as divers with informal ties to Chinese exporters. The interviews did not capture how many these divers were or what their livelihood was while not diving. All interviewed divers resident in urban areas traded catch directly with Chinese exporters, or middlemen employed by the exporter, while divers based in villages traded via middlemen that purchased catch by fishers at landing sites and then sold catch to the exporters in the urban areas. In Mkokotoni and Fumba, the divers traded with middlemen that travelled from Mtoni. These were different middlemen than the gleaning or breath hold fishers traded with. Among exporters and middlemen there were implicit agreements that certain companies traded with certain species and fishers. The divers' equipment needs (tanks, fins, compressors, regulators, boats and engines) had created a rental market controlled by middlemen or exporters.

**Table 2:** The scuba diving operation.

Site	No. Divers	Trade actors	No. Compressors	Processing facilities	Operation
Mtoni	80–100	>4 Exporters	9–10	Exporters have sophisticated boiling and drying facilities	Mtoni was the center of the operation with several resident exporters that own equipment. Divers traded directly with exporters that had developed patron-client relationships with dive teams
Mazizini	40–60	2 Exporters, 3 middlemen that rent out equipment	1–2	Boiling near landing sites and drying at exporters facilities	Many divers were resident in a permanent fishing camp. Local middlemen only traded with equipment were present. All catch was sold directly to exporters. Patron-client relationships with divers “belonging” to certain middlemen.
Mkokotoni	15–20	3 Local middlemen, 1 middleman from Mtoni	2	Two areas for boiling but drying was done at exporters facility in Mtoni	Divers rented equipment and filled tanks via local middlemen. All catch was traded with the middleman from Mtoni. Local middlemen do not trade with scuba diving fishers.
Matemwe	∼10	3 Local middlemen	0	One boiling area	Tanks were usually filled in Mkokotoni. Some equipment owned by middleman in Mkokotoni. Middlemen sold boiled products in Mtoni.
Fumba	<10	1 Local middleman but most catch is taken to Mtoni or Mazizini	0	One boiling area	Village mostly used as an operating village during high season (December). Divers traveled to Fumba to operate from here. Equipment supplied via middlemen and exporters in Mtoni and Mazizini.
Uroa	<10	Catch and equipment is traded with actors in Mtoni	0	Boiling done in village or near temporary camps	Divers from Mtoni or Mazizini operated from Uroa when weather was favorable. Catch was traded directly with exporters and equipment owners.
Chwaka	<10	Catch and equipment is traded with actors in Mtoni	0	Boiling done in village or near temporary camps	Divers from Mtoni or Mazizini operated from Chwaka when weather was favorable. Catch was traded directly with exporters and equipment owners.

Size, structure and description of local operation of the fishery comprised of 200–300 scuba divers in the seven studied villages in Zanzibar.

**Figure 3:**
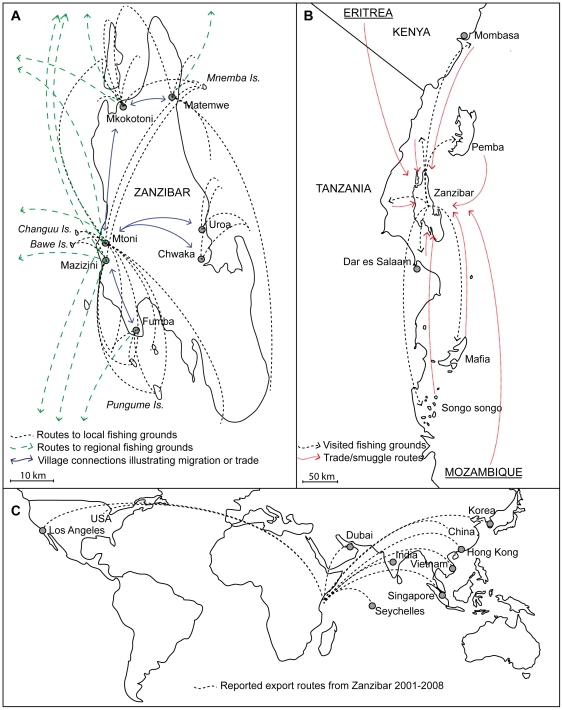
Spatial scale of the scuba diving operation and trade in Zanzibar. a) Local routes of operation with point of origin for both shorter and longer (regional) fishing voyages, b) regional geographic scales of operation for fishing voyages and reported areas where exporters in Zanzibar receive catch or products from, c) reported export destinations from exports during 2001–2008.

The divers reported traveling to many locations to harvest mainly sea cucumbers, at estimated depths down to 50 meters. Locations ranged from nearby fishing grounds along the coast of Zanzibar to distant fishing grounds along mainland Africa (Fig. 3a, b). Divers told narratives of harvesting voyages as far north as to Mombasa in Kenya and as far south as Songo Songo on Tanzania mainland. Both locations are approximately 250 km away from the Mtoni area in Zanzibar. The high mobility and expansion was a result of the reduced profitability of the operation when focusing on nearby areas, which had become depleted. None of the interviewed fishers showed any indication of wanting to reduce effort or applying control on the activity. The local trade network also received sea cucumbers from other areas in the region (Fig. 3b). For example, smuggled catch from Tanzania (where fishing is illegal [39]), was on two occasions presented to the researchers at the exporters location in Mtoni. These were boiled and semi-dried *Holothuria scabra* that had been collected near Dar-es-Salaam and sailed across the Zanzibar channel in a Dhow (local boat) over night. Trade networks included Kenya (also mentioned in [40]), Mozambique (also mentioned in [41]), and Eritrea where illegal harvests seem frequent [42].

Export recorded by DFMR, and indicated by exporters themselves, showed that the fishery directly supports Asian markets (Hong Kong, Singapore, Vietnam, Korea, India and China), North American markets (Los Angeles) and more proximal markets that are probably transits for re-export to Asia (Dubai and Seychelles) (Fig. 3c).

### Catch and economic patterns

A total of 17 sea cucumber species were reported or observed in divers catch and the list of species reflects a diverse range of fishing habitats (Table 3). Divers explicitly mainly targeted sea cucumbers, but also a range of other species suitable to harvest with scuba gear, such as lobster (*Panulirus* spp.), gastropods (*Chicoreus ramosus* and *Pleuroploca trapezium*), octopus (*Octopus cyanea*) and large fish (e.g., *Scaridae* spp. *Serranidae* spp.) using illegal spearguns. Lobster was sold to the local hotels and restaurants. Gastropods are cherished local food and the shells are popular for curio-trade to tourists. Octopus and large fish were sold to both restaurants and local food markets.

**Table 3:** Catch composition and value.

Scientific name	Habitat	Value range per piece (TZS)	Value category
**Sea cucumbers**			
*Holothuria fuscogilva*	Reef (deep)	10,000–20,000	High
*H. nobilis*	Reef (reef flat)	10,000–20,000	High
*H.* sp. “pentard”	Reef (reef front)	10,000–20,000	High
*H. lessoni*	Lagoon (sand)	7,000–9,000	High
*H. scabra*	Lagoon (sand)	5,000–6,000	High
*Thelenota ananas*	Reef (deep)	4,000–8,000	High
*T.anax*	Reef (deep)	1,000–2,000	Medium
*Actinopyga miliaris*	Reef (coral)	1,500	Medium
*Bohadschia atra*	Reef (reef flat)	500–1,000	Medium
*B. vitiensis*	Lagoon (sand)	500–1,000	Medium
*Stichopus monotuberculatus*	Reef (reef flat)	350	Low
*S. herrmanni*	Reef (reef flat)	270	Low
*S. naso*	Reef (back reef)	270	Low
*B. maculisparsa*	Lagoon (sand)	200	Low
*B. subrubra*	Reef (reef flat)	200	Low
*H. isuga*	Lagoon (sand)	200	Low
*H. spinifera*	Lagoon (sand)	150–200	Low
**Other species**			
*Panulirus* spp. (Lobster)	Reef	15,000–20,000	High
Octopus cyanea	Reef	2,500	Medium
Fish (e.g., *Scaridae, Serranidae*)	Reef/Lagoon	1,500	Medium
Ray	Reef/Lagoon	1,500	Medium
*Chicoreus ramosus*	Reef/Lagoon	350	Low
*Pleuroploca trapezium*	Reef/Lagoon	350	Low

Sea cucumbers and other species recorded in scuba divers catch in Zanzibar, listed in descending order of commercial value. Habitat is derived from observation in survey by Eriksson et al (2010) and through interviews with fishers. Value range is recorded price paid to fisher by middleman at point of landing. For fish and ray these are estimated average price per piece, however, this varies greatly between species and size. Category indicates the recorded value range per species. Note that these species were recorded in scuba divers catch and that the island-wide artisanal fishery include more species and sometimes at different prices as outlined by Eriksson et al. (2010). (1 USD = 1,600 TZS, December 2011).

**Figure 4:**
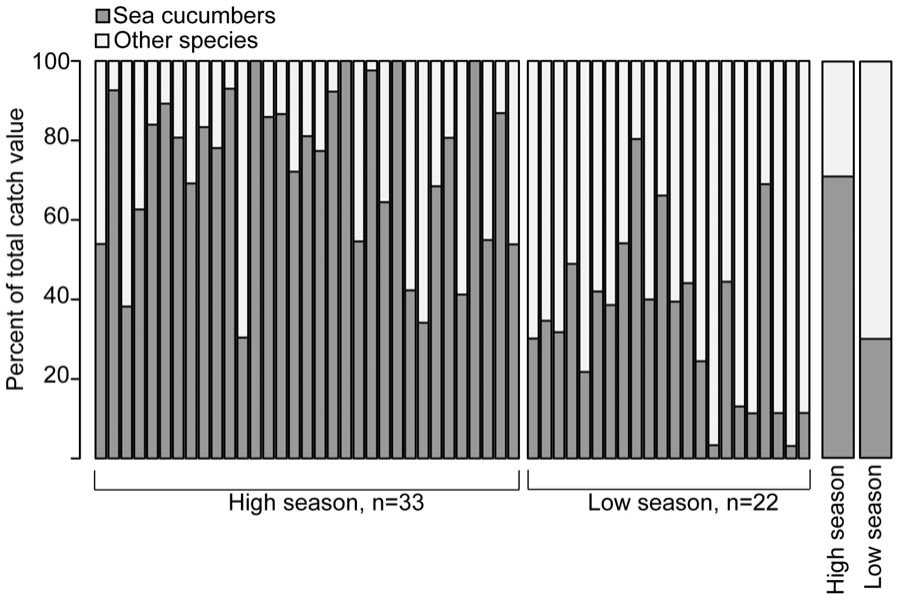
Distribution of catch value between target species for high and low season. Sea cucumbers and other target species contribution to total catch value for trips undertaken during high (December) and low (May) season by the five monitored boats in the Zanzibar scuba diving fishery. n = number of trips during each season.

The total value of all catch from the monitored boats was 4,717,380 Tanzania Shilling (TZS) (1 US Dollar = 1,600 TZS, December 2011), yielding a mean catch value (±SE) of 85,771 TZS (±5,299; n = 55) per boat and trip. This is the value of the first step in the trade-chain, as paid to fishers at point of landing at the beach. The total cost per fishing trip was estimated at 60,000 TSZ; this included rental of all equipment needed for the team (e.g., boat, tanks, regulator, engine). The variability in catch value was best explained by season (63%), while boat or fishing ground were not significant variables with a smaller percentage of explanatory power (1–15%) (Table 4). In high season, the mean (±SE) catch value per boat was 97,469 TSZ (±6,858; n = 33), while in low season it was 68,233 TSZ (±6,972; n = 22). During high season sea cucumbers constituted 72% of the total catch value, while during low season the ratio was reversed with other species contributing to a majority (70%) of catch value (Fig. 4). Following the variability of catch value, the net income of the activity also varied, ranging from a profit of 19,893 TZS to a deficit of −4,987 TZS per team member (Table 5), calculated on the median of seven team members per boat. When the catch value was low, the trade actors used the rented equipment to adjust profits allowing fishers to loan equipment to pay back on days when catch value was higher – a sort of informal institution tying divers to equipment owners (middlemen and exporters) and reinforcing dependence. Divers told narratives of harvesting voyages from when sea cucumber populations were abundant and illustrated a “gold rush” situation. Compared to the incomes in Zanzibar reported by de la Torre-Castro and Rönnbäck [43], the average net income per diver and day during high season was two and half times that of the gross income of net fishers, almost double that of dema (trap) fishers, and the highest recorded net income was ten times the gross income of dragnet fishers.

**Table 4:** Goodness of fit measures.

Variable	Independent contribution (%)	Z-score
Season	63	6.26 *
Boat 1	1	−0.78
Boat 2	1	−0.70
Boat 3	4	−0.15
Boat 4	2	−0.54
Boat 5	2	−0.54
Changuu	7	−0.14
Pungume	5	−0.38
Bawe	15	0.91

Hierarchical partitioning of predictor variables (season, boats, and fishing grounds), illustrating their independent contribution on the variability of catch value among monitored boats, operated by scuba diving teams in Zanzibar. Star represents statistical significance (p<0.05) of the computed Z-value analyzed with a randomization test (1,000 permutations) for hierarchical partitioning. Data are from monitoring of 55 trips.

**Table 5:** Catch value and income for the scuba diving fishers.

Season	Catch value	Net income per boat	Net income per diver
**High season**			
Max	199,250	139,250	19,893
Mean	97,469	37,469	5,353
Min	34,900	−25,100	−3,586
**Low season**			
Max	153,550	93,550	13,364
Mean	68,223	8,223	1,175
Low	25,090	−34,910	−4,987

Catch values and net income for fishing trips undertaken by scuba diving fishing teams during high (December) and low (May) season to three locations (Bawe, Changuu, and Pungume) in Zanzibar. The estimated total cost for the team, 60,000 TSZ, is deducted from the catch value. The net profit per diver is calculated based on an average of seven members per fishing team and boat. (1 USD = 1,600 TZS, December 2011).

**Figure 5:**
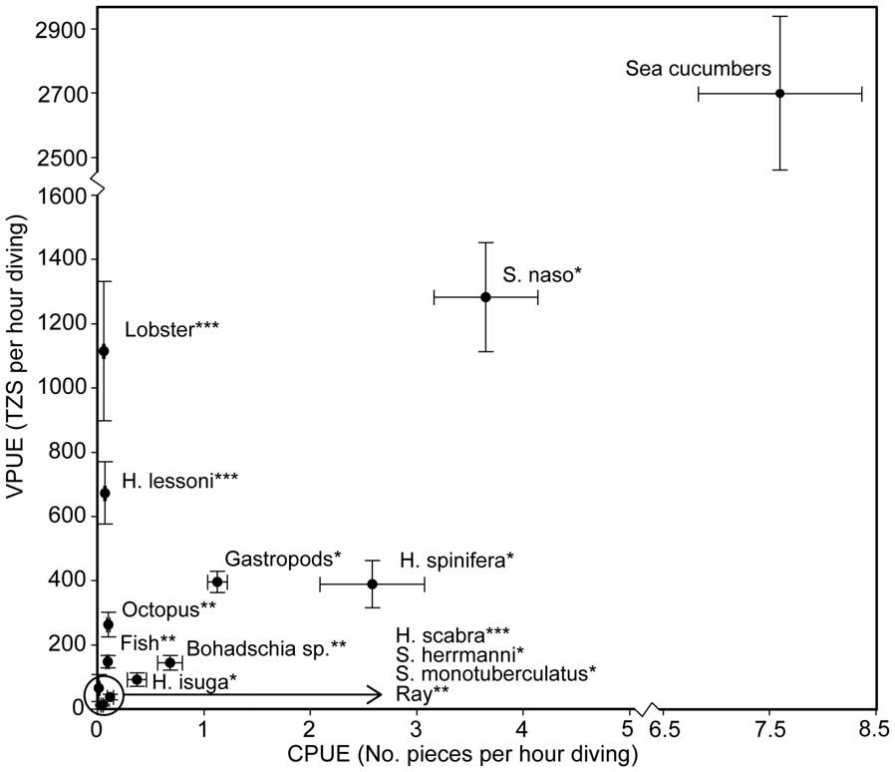
Catch and value per unit effort. Plot of the species-specific mean catch per unit effort (CPUE: in pieces of catch per trip) and value per unit effort (VPUE: TSZ per trip) for 55 trips undertaken by scuba diving fishing teams during high (December) and low (May) season in Zanzibar. Whiskers are SE confidence intervals. Diving time per trip was between 4–4.5 hours and the number of divers per trip was 4–5. Stars indicate per-piece commercial value where; * = Low value ** = Medium value *** = High value. Data point “sea cucumbers” is the pooled CPUE and VPUE for all sea cucumber species.

The VPUE (±SE) of all sea cucumbers species was 2,690 (±257) TSZ per trip (n = 55). As a result of their high per piece price the golden sandfish (*Holothuria lessoni*) and lobster yield high VPUE but still low CPUE (Fig. 5). The highest observed VPUE was for the low value species *Stichopus naso* closely followed by the high valued lobster. They are however on opposite ends on the CPUE scale.

### Social aspects

Divers emphasized that they were involved in the fishery due to the lack of other alternatives, often fishing every day except Fridays, which is the Muslim day for prayers. The diving teams were organized in small groups that worked together to share costs (Table 6). Profit was either shared in the team or kept by the individual fisher. Perceived limiting factors of the activity included physical health issues, depleted target stocks and competition for equipment during the favorable fishing season in December. Health issues were mentioned by the fishers in all focus group interviews. These included common diving related problems, such as the bends and paralysis, but also headaches, bleeding noses and ears. All divers were aware of the risks of diving. In interviews, both a high-risk perception and a more complacent attitude were noted. The importance of learning from friends to avoid injuries was emphasized in this context. Diving in pairs, however, was never mentioned as practice, perhaps as this reduces the area that can be covered while fishing and adds competition between the divers. It is difficult to estimate the actual number of accidents or deaths and no statistics are available, but following our personal observations, and the answers by divers, accidents appeared frequent. One case of death was reported to have occurred during the study. The doctor operating the decompression chamber argued that a major reason for divers getting decompression sickness is because they lack depth gauges. The state of the equipment was not quantified in this study, but from observations, they were often worn out and maintenance was restricted by the rural location of some operations. In Mazizini for example, upon inspection the tanks had new cylinder valves but they were filled with air from a compressor placed under a tree on the beach without a filter on the intake, causing problematic corrosion inside. Profits of the activity were either used to improve the living situation of the individual diver, by building houses and promoting family stability, or to purchase “high-status” items, such as mobile phones, television, mopeds and restaurant meals. The divers had not gone through training and did not use dive tables. Instead, the divers learned from experience and each other.

**Table 6:** Fishing teams organization and social aspects of the scuba diving fishers.

Aspect	Description
Work organization	- Worked in groups (4–10) sharing both costs for equipment and the profits of the catch
	- Worked in groups (4–10) sharing costs for equipment but keep individual catch to themselves
Limiting factors	- Physical limitations (age, strength and health)
	- Poor status of stocks with low abundance of sea cucumbers
	- During high season there was a shortage of equipment to rent (mostly tanks)
Health problems	- Bleeding of nose and ears was common
	- Chronic headache was common
	- Light paralysis of limbs occurred daily within group of divers
	- Several serious cases of paralysis reported every year (some permanently disabled)
	- Death was not uncommon
Risk perception	- The divers were aware of the risks and it was emphasized that learning from each other avoids accidents
	- The divers considered diving normal exercise with light paralysis considered daily routine and nothing to worry about
Use of income	- Many divers were youngsters (<22 years) that used their income for pleasure (restaurants, food and drink) and lifestyle items (mobile phones and television)
	- Divers also used their income for their family and to build good quality houses

Aspects relating to the scuba divers organization and perceptions of the activity as surfaced during interviews and participatory observations in the studied villages in Zanzibar.

## Discussion

### The sea cucumber fishery's' split personality

With this analysis we extend the study by Eriksson et al. [28] to conclude that the sea cucumber fishery in Zanzibar is in fact split into two distinct segments based on the mode of operation and trade organization – the village based artisanal near-shore segment and the more industrialized mobile roving bandit style scuba diving segment. While the breath-hold divers often operate from boats they are not as mobile and cannot harvest at the same high effort or depth as scuba divers can [28]. They also normally trade with the same middlemen as the village gleaners do. Hence, we consider them as part of the artisanal segment. Although the two segments of the fishery are inter-connected as the products transit via the same exporters, this definition of the two segments is crucial because of their different management requirements. Studies on roving bandits in fisheries have normally addressed global scales [7], [10], [21], however, our results show that roving dynamics takes place at considerably smaller spatial scales embedded in the global scale trade. Despite the smaller spatial scale, the management challenges to address such mobility are still substantial. For example, at the local level the divers impact the artisanal segment of the fishery by targeting other villages' fishing grounds, illustrating a loss of resource equity as the mobile divers can evade local management and social control through their mobility. While reformed local management may suffice for improving the situation of the artisanal segment of the fishery [28], navigating available recommendations [19], [29], [30], [44], aspects of spatial scales and different actors must be considered for the industrialized scuba diving segment.

Enforcement capacity is a barrier to improved sea cucumber fisheries [19]. However, we argue that limited capacity extends beyond enforcement, as no relevant management measures are being developed as a response to the situation in Zanzibar. This in turn indicates that current institutions do not match the spatial scale of the operation, a previously documented problem in resource governance [45]–[47], and that international cooperative approaches are needed. A major knowledge gap to defining the full scale of the operation, and managing the trade, is the lack of local capacity to monitor [48], and the current limited knowledge of trade networks in the region. For example, the FAO fishery statistics database does not contain any bêche-de-mer exports from Zanzibar [49], despite Zanzibar's seemingly important role as a link in the regional trade. Given the structure of the diving operation we argue that dialog and cooperation through regional governance partnerships is crucial for improved understanding and management. Although there are organizations in the region that have taken a strong position in understanding the resource and fishery (e.g., The Western Indian Ocean Marine Science Association, WIOMSA) [50], we are concerned to what extent there are any international institutions with mandate for decision-making, or with an agenda to push new policy, that can drive collective participation in the governance of regional fishery operations such as this one.

### International cooperative efforts needed

Foreign exporters utilize the lack of control in Zanzibar to operate this fishery, which seems to have followed a route similar to the southern ocean IUU operation for pelagic fish illustrated by Österblom et al. [10], where institutions are evaded by utilizing weak states that lack enforcement to control their operation. The expansion and mobility of the scuba diving fishery undermines local management in the region, as scuba diving for sea cucumbers is illegal in Kenya [51], and it is illegal to collect sea cucumber in Tanzania since 2006 [39]. Clandestine sea cucumber fishing operations are not unique to Zanzibar. For example, the Chagos archipelago has been a fishing ground for illegal Sri Lankan operations for at least a decade [52]. Also, in Northwest Madagascar scuba diving operations, similar to those described in this study, seem to have become systematic and target for example the French territories îles Glorieuses [31]. Encouragingly, governance of the southern ocean IUU fishery has been improved through international cooperative efforts and diversity of actors [53]. While sea cucumbers have not gained nearly the same attention on the international arena, the southern ocean case may provide a reference for how the situation could be improved through international cooperation, keeping in mind that the capacity to monitor and control differs greatly between East African authorities and that of those involved in the southern ocean. Further research on how similar cooperative efforts can be established to govern this expansive and destructive segment of this fishery would be valuable, along with increased understanding of international institutions that supports collaborative management and ecosystem stewardship.

### Opportunism – patterns and processes in exploitation

The harvesting strategy among the monitored diving teams (boats) of shifting species according to season and availability illustrates that they are opportunistic and resilient to declines in catches. Adaptive responses to environmental change can either amplify or dampen the change [54]. The response to diversify catch illustrates an adaptation that has amplified pressure on the resource and constitutes a destructive conduct. Primarily, it has upheld profitability in the diving trips, generating an amplifying feedback by supporting continuous fishing despite a deteriorated resource base. The broadening of target resources has also added high-effort fishing pressure on other resources than sea cucumbers, broadening the ecological impact of the fishery. The inversed market value - abundance pattern in catch illustrated that, to the divers, abundant low-value species are equally important as scarce high-value species are. This captures how the market also creates an amplifying feedback on the fishery causing a downward spiral in resource abundance. Unless intervened, the market will most probably continue to adjust profit levels, as seen in Papua New Guinea (PNG), where domestic prices for some sea cucumber species increased with over 1,000% from 1991 to 2004 [55]. The fishery in PNG continues at catch rates as low as one animal per ten hours fishing due to lack of income alternatives and the adjusted prices [56]. Therefore, market regulations primarily targeting exporters at the local level should be a focal point for control.

### Trading off health for potential profits

The profits among the fishers, although variable and difficult to determine due to informal agreements for costs, were on occasions comparatively high. Underlying reasons to become a diver were related to this relatively high profit-potential in the context of lack of income alternatives – also illustrated by how divers have traded off health for profits. This is an important aspect to hinge regulative institutions on, because dampening responses to resource degradation, such as reduction in fishing effort, is linked to having options, i.e., to the socioeconomic condition of the fisher [57]. On the other hand, economic poverty with lack of options and a deteriorated resource base may instead trap the system in an undesirable state with self-reinforcing feedback, which requires integrated management approaches and/or external support to escape [58], [59]. Although this study did not quantify the household characteristics of the individual diver, it is clear that the social context (lack of alternatives and economic poverty) is an amplifying feedback on the destructive conduct of this fishery. Solutions to escape such traps should focus on identifying and breaking the feedbacks that keep the system on its current destructive path. Similarly to Purcell et al. [19], we argue that an improvement of the current situation is restricted by the general societal context and that this must be taken into account through holistic approaches in transformations of governance and management.

### Lost equity and independence

That young men suffer dangerous health problems from this activity under uncontrolled circumstances is disheartening and the activity is in great need of intervention, not only from an ecological sustainability perspective, but also from a health and safety perspective. Disadvantageous patron-client relationships seems to be an inherent problem in this fishery, as similar arrangements were in place hundreds of years ago in the central Indo-Pacific [15], [18]. We argue, however, that in Zanzibar such arrangements have increased with the depletion of the fishery and removed profitability from the fisher. As a result of credits, fishers were obliged to continue fishing for the credit provider, trapping them in a dangerous activity, at which stage the potential for “big profits” most likely is gone, as paying back credit will level out high profit fishing trips. That middlemen tie fishers to their enterprise in this manner is without doubt a deliberate strategy and ensures loyalty (future catch) in return for the credit. While the expansive fishing fits with the roving bandits definition, in general, the divers themselves can hardly be called bandits because of their socioeconomic situation and dependence position against trade actors. That the trade is driven by what seems a minimal ambition to maintain profits and health at a fisher level highlights its injustice and undermines opportunities for achieving human rights and therefore also management involvement potential among resource users [60].

## Supporting Information

Text S1 **Interview guide for scuba divers study.** Guide used in focus group interviews and in-depth interviews with respondents in Zanzibar.
(DOC)
