# Supplementary material for: Mobility, Expansion and Management of a Multi-Species Scuba Diving Fishery in East Africa
Source: PLoS One. 2012 Apr 17;7(4):e35504. doi: 10.1371/journal.pone.0035504 (PMC3328463; doi:10.1371/journal.pone.0035504)
Supplement: Text S1 — Interview guide for scuba divers study. Guide used in focus group interviews and in-depth interviews with respondents in Zanzibar. (DOC) [file pone.0035504.s001.doc]

**TEXT S1. FOCUS GROUP INTERVIEWS, DIVERS STUDY**

1. **General information**

Date:

Name of the village:

Name of the interviewer:

Name of the key informants (optional):

Number of key informants:

Types of key informants: a) Divers b) Middleman c) Processor d) Old fisherman e) Monitoring agent f) Other

1. **Interview introduction**

Personal presentation, explain the objective of the visit and research, explain anonymity, explain the use of the results for scientific purposes and better management only.

Prompt phrase “Thank you very much for coming, please tell me about the sea cucumber diving activities in this village”

1. **Themes that should be covered during the discussion/conversation** (not necessarily in that order). Note: If the theme is not brought up by the key informants, please bring it up by using follow up questions).
2. Actors in the fishery (middleman, processors, exporters, divers, snorkelers, etc.)
3. Estimated number of divers in the village in low and high seasons
4. Other villages performing this activity
5. Sea cucumber species found in this site
6. Target species by the divers (sea cucumbers and/ or other marine organisms)
7. Catch trends in the sea cucumber fishery
8. Income estimation per capita and use of revenues
9. Organization (fishing alone or in group, division and distribution of revenues, frequency of diving, depth of diving, narrate the activities in a diving day, etc.)
10. Equipment issues (describe the equipment found in the village, tanks used, cost of tanks)
11. Equipment ownership, links and relationship to the middlemen
12. Mobility (fishing in that village only and/or fishing in nearby villages and/or fishing in other countries)
13. Limiting factors of the sea cucumber diving activity
14. Health related issues (health problems, risks, perceptions and opinions, common accidents reported, associated costs)
15. General problems of the activity (Pros and Cons)
16. General comments
